# Sodium-Glucose Cotransporter-2 (SGLT-2) Inhibitors in Heart Failure: An Umbrella Review

**DOI:** 10.7759/cureus.42113

**Published:** 2023-07-19

**Authors:** Raj Roy, Saketh Vinjamuri, Rishabh Baskara Salian, Nosheen Hafeez, Dakshin Meenashi Sundaram, Tirath Patel, Thulasi Ram Gudi, Advait M Vasavada

**Affiliations:** 1 Internal Medicine, Gandhi Medical College and Hospital, Secunderabad, IND; 2 Internal Medicine, Kasturba Medical College, Manipal, IND; 3 Medicine, King Edward Medical University, Lahore, PAK; 4 Internal Medicine, Employees State Insurance Corporation (ESIC) Medical College and Post Graduate Institute of Medical Sciences and Research (PGIMSR), Chennai, IND; 5 Surgery, American University of Antigua, St. John, ATG; 6 Internal Medicine, Merit Health River Region, Vicksburg, USA; 7 Internal Medicine, M. P. Shah Medical College, Jamnagar, IND

**Keywords:** heart failure prognosis, cardiology research, heart failure hospitalization, sodium-glucose cotransporter-2 (sglt-2) inhibitors, sglt2 inhibitors and heart failure

## Abstract

Heart failure remains a leading cause of hospitalization and death, and presents a significant challenge for healthcare providers despite the advancements in its management. This umbrella review aimed to pool the results of meta-analyses on the use of sodium-glucose cotransporter-2 (SGLT-2) inhibitors in the treatment of heart failure patients. A literature search was done on five databases: PubMed, Cochrane Library, Scopus, Global Index Medicus, and Science Direct for articles with full texts available online. Meta-analyses of five or more randomized controlled trials (RCTs) were included; the assessment of multiple systematic reviews (AMSTAR) was used to assess the quality of included studies. A systematic search identified 10 relevant meta-analyses of RCTs, with primary analyses including outcome data from 171,556 heart failure patients. A pooled review showed that SGLT-2 inhibitors significantly reduced the risk of heart failure hospitalization, cardiovascular death, mortality, serious adverse events, and improved quality of life. SGLT-2 inhibitors are likely safe and effective in managing patients with heart failure especially considering the acute outcomes.

## Introduction and background

Heart failure is a chronic ailment that is characterized by the heart's inability to pump blood efficiently, and some of its symptoms are fatigue, shortness of breath, and fluid retention in the body [1]. With its multifaceted nature and widespread impact on individuals worldwide, heart failure poses significant challenges to hospital length of stay, death, and morbidity. As a chronic and progressive ailment, it not only compromises the quality of life in those affected but also places a substantial burden on healthcare systems worldwide [2]. To address this issue, a multidisciplinary approach is necessary that includes both pharmacological and non-pharmacological interventions [3,4].

In addition to its advantages for treating type 2 diabetes, sodium-glucose cotransporter 2 (SGLT-2) inhibitors have recently become a promising therapeutic option for treating patients with heart failure [4]. SGLT-2 inhibitors are oral anti-diabetic drugs that reduce the kidneys' ability to reabsorb glucose, thereby increasing the excretion of glucose [5]. SGLT-2 inhibitors were initially intended to lower blood glucose levels in patients with type 2 diabetes but demonstrated additional effects beyond their primary role. In the studies investigating their efficacy, researchers noticed a significant reduction in cardiovascular events and heart failure hospitalizations among those treated with SGLT-2 inhibitors compared to traditional diabetes medications. This unexpected finding sparked curiosity and launched a series of investigations aimed at unraveling the mechanisms behind these remarkable outcomes [6-9]. There have been several meta-analyses to evaluate the efficiency and safety of SGLT-2 inhibitors in patients with heart failure and the results from these studies have been conflicting, indicating a need for further discovery [6-9].

The objective of this umbrella review is to summarize the results of meta-analyses on the use of SGLT-2 inhibitors in the treatment of heart failure patients and to offer a comprehensive evaluation of the efficacy and safety of these drugs in this population. This review provides a thorough summary of the available research and is helpful to policymakers and healthcare professionals in making decisions about the use of SGLT-2 inhibitors in the treatment of heart failure.

## Review

Methods

The Preferred Reporting Items for Systematic Reviews and Meta-Analyses (PRISMA) guidelines, according to Liberati et al., were followed while conducting this study [10].

Search methods

The literature was searched for topic-related articles using five databases: PubMed, Cochrane Library, Scopus, Global Index Medicus, and Science Direct looking for the search terms like sodium glucose co-transporter-2, SGLT-2, heart failure, and cardiac failure. Additional keywords and the full search strategies are outlined in Table 1. The search was performed for articles published from inception till January 30, 2023, and the type of studies to be found were restricted to systematic reviews with meta-analyses.

**Table 1:** Search strings used for the umbrella review

Database	Search string
ScienceDirect	("SGLT-2 inhibitor*" OR "sodium-glucose cotransporter 2 inhibitor*" OR "dapagliflozin" OR "empagliflozin" OR "canagliflozin" OR "ertugliflozin" OR "ipragliflozin" OR "luseogliflozin") AND ("heart failure" OR "cardiac failure" OR "congestive heart failure" OR "CHF" OR "left ventricular dysfunction")
PubMed	Filters applied: Review, Search: (("Sodium-Glucose Transport Proteins, Type 2" [MeSH Terms] OR "sodium-glucose cotransporter 2 inhibitor*" [Title/Abstract] OR "dapagliflozin" [Title/Abstract] OR "empagliflozin" [Title/Abstract] OR "canagliflozin" [Title/Abstract] OR "ertugliflozin" [Title/Abstract] OR "ipragliflozin" [Title/Abstract] OR "luseogliflozin" [Title/Abstract])) AND (("Heart Failure" [MeSH Terms] OR "heart failure" [Title/Abstract] OR "cardiac failure" [Title/Abstract] OR "congestive heart failure" [Title/Abstract] OR "CHF" [Title/Abstract] OR "left ventricular dysfunction" [Title/Abstract]))
Global Index Medicus	("SGLT-2 inhibitor*" OR "sodium-glucose cotransporter 2 inhibitor*" OR "dapagliflozin" OR "empagliflozin" OR "canagliflozin" OR "ertugliflozin" OR "ipragliflozin" OR "luseogliflozin") AND ("heart failure" OR "cardiac failure" OR "congestive heart failure" OR "CHF" OR "left ventricular dysfunction"). Additional keywords used for title screening: Systematic review, meta-analysis,
Scopus	TITLE (("SGLT-2 inhibitor*" OR "sodium-glucose cotransporter 2 inhibitor*" OR "dapagliflozin" OR "empagliflozin" OR "canagliflozin" OR "ertugliflozin" OR "ipragliflozin" OR "luseogliflozin") AND ("heart failure" OR "cardiac failure" OR "congestive heart failure" OR "CHF" OR "left ventricular dysfunction")) AND (LIMIT-TO (DOCTYPE, “re”))
Cochrane library	#1 ("SGLT-2 inhibitor*" or "sodium-glucose cotransporter 2 inhibitor*" or "dapagliflozin" or "empagliflozin" or "canagliflozin" or "ertugliflozin" or "ipragliflozin" or "remogliflozin" or "tofogliflozin" or "luseogliflozin"):ti,ab,kw #2 ("heart failure" or "cardiac failure" or "congestive heart failure" or "CHF" or "left ventricular dysfunction"):ti,ab,kw #3 #1 AND #2

Eligibility criteria

The criteria for inclusion of a study were as follows: systematic review and meta-analysis (SR-MA) studies, SR-MAs with atleast five or more randomized controlled trials (RCTs), and papers in the English language. Given the amount of literature available on the topic, we determined that a meta-analysis with at least five studies would help avoid poor-quality SR-MA studies. The criteria for exclusion of the study were as follows: systematic reviews that did not pool data or carry out meta-analysis; narrative reviews; studies of the efficacy or safety of SGLT-2 inhibitors in patients that did not have heart failure; studies that did not compare SGLT-2 inhibitors with placebo and only evaluated individual factors in heart failure patients such as age, diabetes status, gender, or baseline therapy.

With a larger number of primary studies, the statistical power of the analysis increases. This means that the umbrella review can provide more robust and reliable conclusions by pooling data from a larger sample size. The inclusion of SR-MAs with five or more primary studies in an umbrella review will lead to narrower confidence intervals and hence more precision. This increased precision allows for more accurate estimation of the outcomes of patients taking SGLT-2 inhibitors. 

Study selection

Two authors independently assessed the titles and abstracts of papers to determine if they met the inclusion and exclusion criteria. After entering each reference in Endnote software (Version X9; Clarivate Analytics, Pennsylvania), duplicates were eliminated. Every meta-analysis that satisfied the requirements for inclusion had its reference lists manually searched to make sure no pertinent study was overlooked. The entire text was examined in cases when abstracts did not provide enough information to determine if inclusion or exclusion criteria were met. Every meta-analysis that satisfied either set of criteria was carefully evaluated before the final inclusion decision was made, and any differences between the two independent reviewers were settled in a consensus meeting with a third reviewer.

Data extraction

The following data was extracted from each of the included study: the primary author, the journal, the year of publication, the search range in years of studies that were included in the meta-analysis, and the number of primary studies that were included in the meta-analysis. Date range of the primary studies that were part of the meta-analyses were listed along with the following: the sample size of the study, the population it was drawn from, the meta-findings, analysis such as the mean difference and standardized mean difference for the risk ratio, confidence intervals (CI), the p-value, and the classification of heart failure based on ejection fraction. 

Assessment of methodological quality

The internal validity of the meta-analyses included in this research was evaluated using the assessment of multiple systematic reviews (AMSTAR) tool [11]. In a systematic review, AMSTAR rates the accuracy of reporting and methodology. With a kappa score of 0.7 for agreement on individual questions and an interclass correlation coefficient of 0.84, it demonstrated high reliability and validity. The updated version of AMSTAR (v.2) analyzes the quality of systematic reviews, identifies its strengths and weaknesses, and draws attention to the quality of the included studies [12].

Degree of primary study overlap

The corrected covered area (CCA) created by Pieper et al. can be used to quantify overlap in primary research included in reviews [13]. By "dividing the frequency of repeated occurrences of the index publication in other reviews by the product of index publications and reviews, and this product is decreased by the number of index publications," it is able to find index publications. Slight overlap (0-5), moderate overlap (6-10), high overlap (11-15), and very high overlap (>15) are the four categories under which overlap is classified.

Results

Study Selection

**Figure 1:**
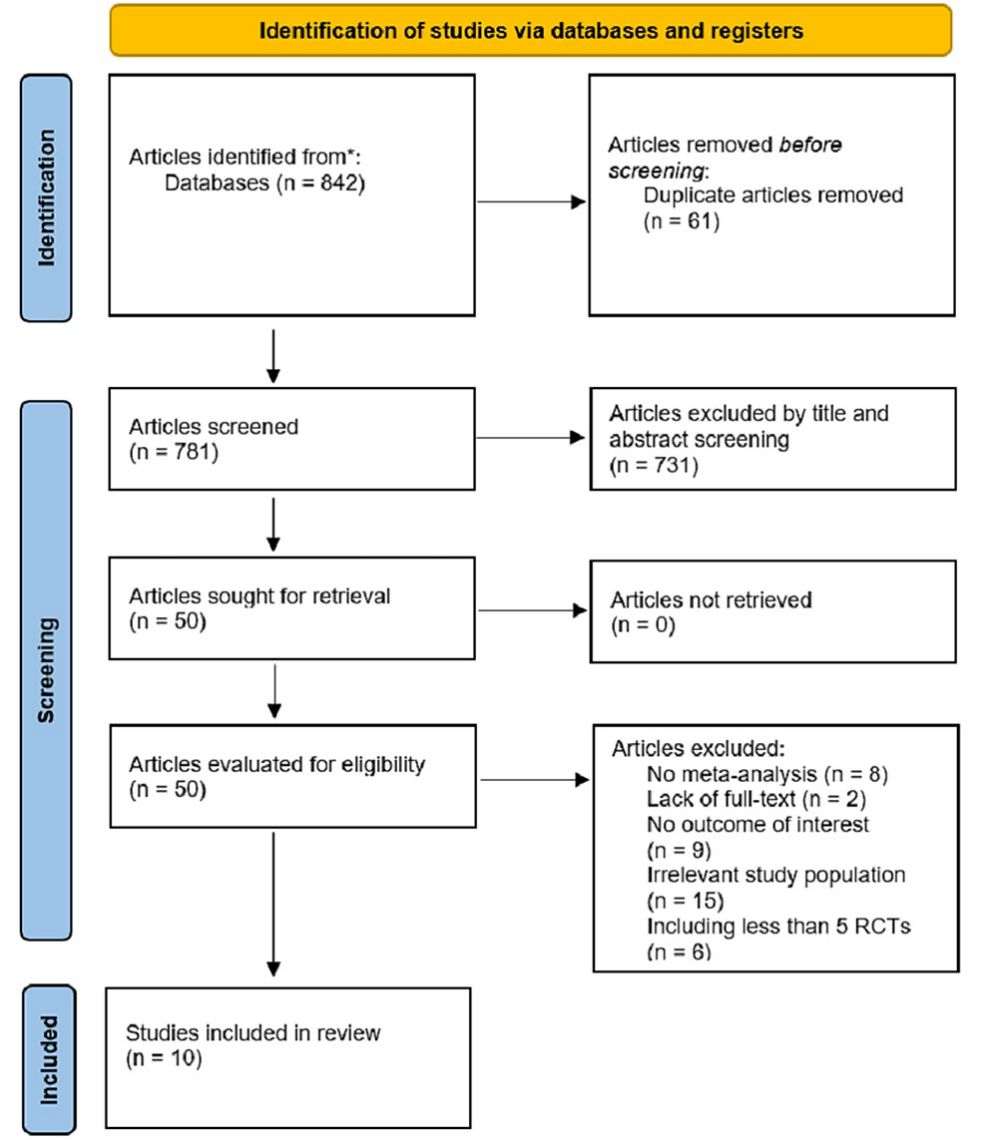
Preferred Reporting Items for Systematic Reviews and Meta-Analyses (PRISMA) chart

The electronic database search provided an initial 841 studies. A manual search of relevant journals and reference lists provided one study. Sixty-one duplicates were removed from the 842, leaving 781 articles from which a title and abstract screening removed 731 as not relevant. The full text of all remaining 50 articles was downloaded to be assessed in detail; 40 of these were excluded in the detailed assessment and 10 meta-analyses were included in the meta-review. Figure 1 shows how the search and exclusion were executed.

Characteristics of Included Studies

Table 2 gives the details of the included meta-analyses’ characteristics. All were published between 2020 and 2022; each was published in a different journal; there were between five and ten studies in the included meta-analyses.

**Table 2:** Main characteristics of included studies [6-9,14-19]. ACM: all cause mortality, WHF: worsening heart failure, HHF: hospitalization for heart failure, HF: heart failure, CVD: cardiovascular death, SGLT-2: sodium-glucose cotransporter 2, KCCQ: Kansas City Cardiomyopathy Questionnaire score, HRQoL: health related quality of life.

Study ID (author and publication year)	Journal	Range of years of included studies	Primary studies (n)	Sample size	Type of heart failure	Findings (Meta-analysis results)
Chen et al., 2021 [14]	Front Endocrinol (Lausanne)	NA-2021	5	36998	not specified	SGLT-2 inhibitors significantly improved the outcome of CV death or HHF (HR 0.69[95%CI, 0.63-0.77], P < 0.001); SGLT-2 inhibitors can significantly reduce the rate of CV death (HR 0.80[95%CI, 0.69-0.92], P = 0.001); SGLT-2 inhibitors can reduce the incidence of HHF (HR 0.67[95%CI, 0.60-0.76], P < 0.001); SGLT-2 inhibitors can reduce the rate of ACM compared with placebo (HR 0.74[95%CI, 0.64-0.86], P < 0.001)
Lu et al., 2021 [15]	European Journal of Internal Medicine	NA-2020	8	16460	not specified	SGLT-2i significantly reduced the risk for CV death or HHF by 23% compared to placebo (HR: 0.77, 95% CI: 0.72–0.82); SGLT-2i was associated with a statistically significant 32% reduction in HHF compared to placebo (HR: 0.68, 95% CI: 0.62–0.75); SGLT-2i led to a 15% reduction in CV death compared to placebo (HR: 0.85, 95% CI: 0.76–0.94); SGLT-2i led a 16% reduction in ACM compared to placebo (HR: 0.84, 95% CI: 0.77–0.92)
Singh & Singh, 2021 [16]	Diabetes & Metabolic Syndrome	NA-2020	9	19741	not specified	SGLT-2i was associated with a significant 26% reduction in composite of CV death or HHF.HR (0.74; 95% CI, 0.69–0.79; p < 0.001); SGLT-2i led to significant reduction in CV death compared to placebo HR (0.86; 95% CI, 0.78–0.95; p = 0.003); SGLT-2 led to 32% significant reduction in HHF compared to placebo (HR 0.68; 95% CI, 0.62–0.74; p < 0.001)
Vaduganathan et al., 2022 [17]	Lancet	2015-2022	5	TOTAL 21 947	not specified	SGLT-2i led to significant 23% reduction in CV death or HHF, HR 0·77 [0·72–0·82]; SGLT-2i led to a significant reduction in CV death,HR 0·87 [0·79–0·95]; SGLT-2i led to significant reduction in HHF HR 0·72 [0·67–0·78]; SGLT-2i led to reduction in ACMHR 0·92 [0·86–0·99]
Li et al., 2021 [9]	International Journal of Cardiology	NA-2020	7	14113	not specified	SGLT2i was associated with lower incidences cardiovascular death or HHF, (RR = 0.773; 95% CI, 0.719–0.831; p < 0.001; I2 = 8.1%); SGLT2i was linked with lower incidences of Cardiovascular death,(RR 0.872; 95% CI, 0.788–0.964; p = 0.008; I2 = 0.0%); SGLT2i was linked with lower incidences of HHF(RR 0.722; 95% CI, 0.657–0.793; p < 0.001; I2 = 15.4%); SGLT2i was linked with serious decrease in renal function(RR 0.673; 95% CI, 0.549–0.825; p < 0.001; I2 = 17.7%); SGLT2i was linked with serious decrease of serious adverse events (RR 0.867; 95% CI, 0.808–0.930; p < 0.001; I2 = 60.1%)
Butler et al., 2020 [18]	ESC Heart Fail	NA-2020	7	16 820	not specified	SGLT2i significantly reduced the risk of HHF or CV death [HR: 0.77 (0.72–0.83); P < 0.001; I 2 = 0%]; SGLT2i significantly reduced the time to first HF hospitalization,[HR: 0.71 (0.64–0.78); P < 0.001; I 2 = 0]; SGLT2i significantly reduced the risk of cardiovascular mortality [HR: 0.87 (0.79–0.96); P = 0.005; I 2 = 0%]; SGLT2i significantly reduced the risk of all‐cause mortality.[HR: 0.89 (0.82–0.96); P = 0.004; I 2 = 0%]
He et al., 2021 [19]	International Journal of Cardiology	NA-2021	9	9428	HFrEF	SGLT-2 led to a significant improvement in HRQoL assessed by (KCCQ),(MD: 2.13, 95% CI: 1.11 to 3.14, p < 0.001); No significant difference was observed in exercise capacity assessed by 6-min walk test distance between SGLT-2 inhibitors and placebo(MD 24.45, 95%CI -22.82 to 71.72, P = 0.31)
Yang et al., 2022 [7]	Frontiers in Cardiovascular Medicine		10	10,334	HFpEF	SGLT2 decreased the incidence of the composite outcome, HR: 0.78, 95% CI: 0.69–0.88, p = 0.00); SGLT2 lowered the incidence of hospitalization for heart failure (OR: 0.71, 95% CI: 0.61–0.83, p = 0.00; I2 = 0.00%, p = 0.970); NO significant difference in CV death between SGLT2 and placebo (OR: 1.02, 95% CI: 0.77–1.35, p = 0.888; I2 = 35.5%, p = 0.212); No advantage in reducing all-cause mortality between SGLT2 and placebo. (OR: 0.99, 95% CI: 0.87–1.13, p = 0.936; I2 = 0.00%, p = 0.973); SGLT2 led to a greater improvement in KCCQ from baseline compared with placebo (MD: 2.74, 95% CI: 1.30–4.18, p = 0.00)
Tsampasian et al., 2021 [6]	European journal of preventive cardiology	NA-2021	5	9726	HFpEF	SGLT2 was associated with a significant reduction in CV death or HHF,(HR = 0.78, 95% CI: 0.69, 0.87; I2 = 0%); SGLT2 was associated with a significant reduction in HHF (HR = 0.71, 95% CI: 0.61, 0.84; I2 = 0%); No significant differences between SGLT2 and placebo on HF patients in terms of CV death (HR = 1.01, 95% CI: 0.80, 1.28; I2= 23%); No significant differences between SGLT2 and placebo on HF patients in terms all-cause mortality(HR = 1.01, 95% CI: 0.89, 1.14; I2 = 0%)
Wang et al., 2022 [8]	European Journal of Medical Research	NA- 2022	6	15989	HFmEF or HFpEF	SGLT2 inhibitors significantly reduced the composite outcome of HF hospitalization or cardiovascular death,[HR: 0.79 (0.72–0.85); I2 = 0%; P < 0.001]; SGLT2 inhibitors significantly reduced HF hospitalizations.[HR: 0.74 (0.67–0.82); I2 = 0%; P < 0.00001]; SGLT2 inhibitors reduced incidence of any serious adverse events compared to placebo[OR: 0.89 (0.83–0.96); I2 = 0%; P = 0.002]

The reviews included a total of 27 primary studies, and the meta-analyses used a minimum of five and a maximum of ten studies (Table 3). No consistent way of including primary studies was used and none of the 27 main studies was cited in all 10 meta-analyses. With a CCA score of 18.1%, the overlap across the primary studies that were considered was very high.

**Table 3:** Citation matrix for the primary studies included in each meta-analysis [6-9,14-19]

Primary study	Li et al., 2021 [9]	Yang et al., 2022 [7]	Wang et al., 2022 [8]	Tsampasian et al., 2021 [6]	Chen et al., 2021 [14]	Butler et al., 2020 [18]	Singh & Singh, 2021 [16]	Lu et al., 2021 [15]	He et al., 2021 [19]	Vaduganathan et al., 2022 [17]
Zinman et al., 2015	⁺					⁺				
Neal et al., 2017	⁺					⁺				
Wiviott et al., 2019	⁺		⁺			⁺				
Nassif et al., 2019	⁺						⁺		⁺	
McMurray et al., 2019	⁺					⁺	⁺		⁺	⁺
Packer et al., 2020	⁺					⁺	⁺	⁺	⁺	⁺
Bhatt, Szarek, Steg, et al., 2020	⁺	⁺	⁺	⁺	⁺	⁺		⁺		⁺
Anker, Butler, Filippatos, Ferreira, et al., 2021		⁺	⁺	⁺						
Kato et al., 2019		⁺		⁺	⁺		⁺	⁺		
Cosentino et al., 2020		⁺		⁺			⁺			
Abraham et al., 2021		⁺							⁺	
Butler et al., 2021		⁺							⁺	
Bhatt, Szarek, Pitt, et al., 2020		⁺	⁺	⁺			⁺			
Nassif et al., 2021		⁺								
Spertus et al., 2022		⁺								
NCT03877224		⁺								
Cannon et al., 2020			⁺			⁺		⁺		
Solomon et al., 2022			⁺							⁺
Rådholm et al., 2018					⁺		⁺	⁺		
Petrie et al., 2020					⁺			⁺		
Anker, Butler, Filippatos, Marx, et al., 2021					⁺					⁺
Fitchett et al., 2017							⁺	⁺		
Kosiborod et al., 2017							⁺		⁺	
Sarraju et al., 2020								⁺		
Santos-Gallego et al., 2021									⁺	
Jensen, et al., 2020									⁺	
Lee et al., 2020									⁺	

Methodological Quality Assessment

AMSTAR 2 rated eight studies as being of moderate quality, one study as being of high quality, and one study as low in quality. While limitations were mentioned, none of the included meta-analyses addressed the possible impact of risks of bias (in their study) in a quantifiable manner; half failed to carry out an adequate investigation of publication bias, while only one of the ten provided a list of excluded studies (Table 4).

**Table 4:** AMSTAR 2 evaluation for included meta-analyses [6-9,14-19] N: no, Y: yes, P.Y: partial yes, AMSTAR: assessment of multiple systematic reviews.

STUDY	AMSTAR 2 Items																FINAL SCORE
	1	2	3	4	5	6	7	8	9	10	11	12	13	14	15	16	
Butler et al., 2020 [18]	Y	Y	Y	Y	Y	Y	N	Y	Y	Y	Y	N	Y	N	N	Y	Moderate
Chen et al., 2021 [14]	Y	Y	Y	P.Y	Y	Y	N	Y	Y	Y	Y	N	Y	Y	N	Y	Moderate
He et al., 2021 [19]	Y	Y	Y	Y	Y	Y	N	Y	Y	Y	Y	N	Y	Y	N	Y	Moderate
Li et al., 2021 [9]	Y	Y	Y	Y	Y	Y	N	Y	Y	Y	Y	N	Y	Y	Y	Y	Moderate
Lu et al., 2021 [15]	Y	Y	Y	Y	Y	Y	N	Y	Y	Y	Y	N	Y	Y	Y	Y	Moderate
Singh & Singh, 2021 [16]	Y	Y	Y	P.Y	Y	Y	Y	Y	Y	Y	Y	N	Y	Y	Y	Y	High
Tsampasian et al., 2021 [6]	Y	Y	Y	Y	Y	Y	N	Y	Y	Y	Y	N	N	Y	Y	Y	Moderate
Vaduganathan et al., 2022 [17]	Y	Y	Y	N	Y	Y	N	Y	Y	Y	Y	N	Y	Y	N	Y	low
Wang et al., 2022 [8]	Y	Y	Y	P.Y	Y	Y	N	Y	Y	Y	Y	N	Y	Y	N	Y	Moderate
Yang et al., 2022 [7]	Y	Y	Y	Y	Y	Y	N	Y	Y	Y	Y	N	Y	Y	N	Y	Moderate

Discussion

In order to demonstrate the efficacy and safety of SGLT-2 inhibitors for patients with heart failure, this study was conducted to provide a systematic aggregation of evidence from several meta-analyses into a single, current database that is easily accessible. Ten meta-analyses were incorporated into the review, and the combined findings of this study showed that SGLT-2 inhibitors are linked to significantly lower rates of cardiovascular death or heart failure hospitalization in patients with heart failure, cardiovascular death, all-cause mortality, and heart failure hospitalization outcomes. However, two meta-analyses revealed no significant difference in cardiovascular death and all-cause mortality outcomes between SGLT-2 inhibitors and placebo [6,7].

Most studies discovered that the use of SGLT-2 inhibitors may result in better outcomes for individuals with heart failure, including decreased rates of hospitalization and death from cardiovascular causes. And since SGLT-2 inhibitors are also successful at controlling glucose levels in people with type 2 diabetes, this evidence will be especially helpful for people with heart failure who also have uncontrolled diabetes. Additionally,looking at most of the studies, SGLT-2 inhibitors were also linked to less serious adverse events and a higher improvement in Kansas City Cardiomyopathy Questionnaire (KCCQ) scores. In comparison to other heart failure medicines, SGLT-2 inhibitors may be associated with enhanced safety due to the decreased frequency of major side events and improvement in KCCQ score. This may result in better outcomes and a higher quality of life for heart failure patients as well as lower hospitalization and other medical intervention expenses [6-9,14-19].

Results also highlight the necessity of compiling the meta-analyses into a single, comprehensive review to reflect the validity of the conclusions reached by each individual meta-analysis. That was the study's objective, and it is what the study ultimately attained. Because reducing cardiovascular death, heart failure hospitalization, and all-cause mortality leads to better performance and lower treatment costs; this work will have significant implications for health organizations as well as individuals with heart failure and medical professionals. This study's findings also suggest that SGLT-2 inhibitors may reduce serious adverse effects in addition to improving the quality of life. Either way, the net result is probably going to be less strain on the healthcare system. One major concern was that no study evaluated participants with advanced renal failure with GFR<20 mL/min/1.73 m2. Hence, it remains questionable whether the drug should be used in this subset of patients. The American College of Cardiology (ACC) expert consensus also has highlighted a similar problem where renal compromised patients may have attenuated effects to the use of this drug [20]. SGLT-2 inhibitors may cause an adverse impact on renal function during the first year of therapy. As the glycosuric effect would be compromised in advanced renal disease, its mechanism of action may be compromised. So, SGLT-2 inhibitors will do more harm than benefit such patients. This area needs active investigation [20-22].

The umbrella review has some limitations; multiple meta-analyses have overlapping primary studies between them (as illustrated in the citation matrix above), so the studies that appear in more reviews (the ones that overlap in multiple studies) could have a stronger impact on the findings of this review. More meta-analyses have been done on earlier published studies, which means they are probably overrepresented in the literature, leading to publication bias.

## Conclusions

Prior to this research, there had not been an umbrella review on the use of SGLT-2 inhibitors in the treatment of patients with heart failure. Overall, our review of the literature shows that SGLT-2 inhibitors can reduce the risk of cardiovascular death, all-cause mortality, and heart failure hospitalization when used in the treatment of heart failure. Most meta-analysis studies support the use of SGLT-2 inhibitors in the treatment of heart failure as SGLT-2 inhibitors are also linked with reduced serious adverse effects; however, its use in patients with severely impaired renal function remains an enigma.

## References

[1] Tsao CW, Aday AW, Almarzooq ZI (2022). Heart disease and stroke statistics—2022 update: a report from the American Heart Association. Circulation.

[2] Taylor CJ, Ordóñez-Mena JM, Roalfe AK, Lay-Flurrie S, Jones NR, Marshall T, Hobbs FD (2019). Trends in survival after a diagnosis of heart failure in the United Kingdom 2000-2017: population based cohort study. BMJ.

[3] McMurray JJ, Packer M, Desai AS (2014). Angiotensin-neprilysin inhibition versus enalapril in heart failure. N Engl J Med.

[4] Singh AK, Singh R (2019). Heart failure hospitalization with SGLT-2 inhibitors: a systematic review and meta-analysis of randomized controlled and observational studies. Expert Rev Clin Pharmacol.

[5] Kosiborod M, Cavender MA, Fu AZ (2017). Lower risk of heart failure and death in patients initiated on sodium-glucose cotransporter-2 inhibitors versus other glucose-lowering drugs. Circulation.

[6] Tsampasian V, Elghazaly H, Chattopadhyay R (2022). Sodium glucose co-transporter 2 inhibitors in heart failure with preserved ejection fraction: a systematic review and meta-analysis. Eur J Prev Cardiol.

[7] Yang D, Zhang Y, Yan J, Liu M, An F (2022). SGLT-2 inhibitors on prognosis and health-related quality of life in patients with heart failure and preserved ejection fraction: a systematic review and meta-analysis. Front Cardiovasc Med.

[8] Wang Y, Gao T, Meng C, Li S, Bi L, Geng Y, Zhang P (2022). Sodium-glucose co-transporter 2 inhibitors in heart failure with mildly reduced or preserved ejection fraction: an updated systematic review and meta-analysis. Eur J Med Res.

[9] Li X, Zhang Q, Zhu L, Wang G, Ge P, Hu A, Sun X (2021). Effects of SGLT2 inhibitors on cardiovascular, renal, and major safety outcomes in heart failure: a meta-analysis of randomized controlled trials. Int J Cardiol.

[10] Liberati A, Altman DG, Tetzlaff J (2009). The PRISMA statement for reporting systematic reviews and meta-analyses of studies that evaluate healthcare interventions: explanation and elaboration. BMJ.

[11] Shea BJ, Grimshaw JM, Wells GA (2007). Development of AMSTAR: a measurement tool to assess the methodological quality of systematic reviews. BMC Med Res Methodol.

[12] Shea BJ, Reeves BC, Wells G (2017). AMSTAR 2: a critical appraisal tool for systematic reviews that include randomised or non-randomised studies of healthcare interventions, or both. BMJ.

[13] Pieper D, Antoine SL, Mathes T, Neugebauer EA, Eikermann M (2014). Systematic review finds overlapping reviews were not mentioned in every other overview. J Clin Epidemiol.

[14] Chen C, Peng H, Li M, Lu X, Huang M, Zeng Y, Dong G (2021). Patients with type 2 diabetes mellitus and heart failure benefit more from sodium-glucose cotransporter 2 inhibitor: a systematic review and meta-analysis. Front Endocrinol (Lausanne).

[15] Lu Y, Li F, Fan Y, Yang Y, Chen M, Xi J (2021). Effect of SGLT-2 inhibitors on cardiovascular outcomes in heart failure patients: a meta-analysis of randomized controlled trials. Eur J Intern Med.

[16] Singh AK, Singh R (2021). Cardiovascular outcomes with SGLT-2 inhibitors in patients with heart failure with or without type 2 diabetes: a systematic review and meta-analysis of randomized controlled trials. Diabetes Metab Syndr.

[17] Vaduganathan M, Docherty KF, Claggett BL (2022). SGLT2 inhibitors in patients with heart failure: a comprehensive meta-analysis of five randomised controlled trials. Lancet.

[18] Butler J, Usman MS, Khan MS (2020). Efficacy and safety of SGLT2 inhibitors in heart failure: systematic review and meta-analysis. ESC Heart Fail.

[19] He Z, Yang L, Nie Y (2021). Effects of SGLT-2 inhibitors on health-related quality of life and exercise capacity in heart failure patients with reduced ejection fraction: a systematic review and meta-analysis. Int J Cardiol.

[20] Maddox TM, Januzzi JL Jr, Allen LA (2021). 2021 update to the 2017 ACC expert consensus decision pathway for optimization of heart failure treatment: answers to 10 pivotal issues about heart failure with reduced ejection fraction: a report of the American College of Cardiology Solution Set Oversight Committee. J Am Coll Cardiol.

[21] Kittleson MM, Panjrath GS, Amancherla K (2023). 2023 ACC expert consensus decision pathway on management of heart failure with preserved ejection fraction: a report of the American College of Cardiology Solution Set Oversight Committee. J Am Coll Cardiol.

[22] Zelniker TA, Braunwald E (2020). Mechanisms of cardiorenal effects of sodium-glucose cotransporter 2 inhibitors: JACC state-of-the-art review. J Am Coll Cardiol.

